# Mitral valve stretched to its limit: a case of an aborted sudden cardiac death with extensive fibrotic ventricular remodelling

**DOI:** 10.1093/ehjci/jeaf063

**Published:** 2025-02-24

**Authors:** Philippe J van Rosendael, P Stefan Biesbroek, Timion A Meijs, Marco J W Gotte, Luuk H G A Hopman

**Affiliations:** Department of Cardiology, Amsterdam UMC, De Boelelaan 1118, Amsterdam 1081 HV, The Netherlands; Department of Cardiology, Amsterdam UMC, De Boelelaan 1118, Amsterdam 1081 HV, The Netherlands; Department of Cardiology, Amsterdam UMC, De Boelelaan 1118, Amsterdam 1081 HV, The Netherlands; Department of Cardiology, Amsterdam UMC, De Boelelaan 1118, Amsterdam 1081 HV, The Netherlands; Department of Cardiology, Amsterdam UMC, De Boelelaan 1118, Amsterdam 1081 HV, The Netherlands

**Figure:**
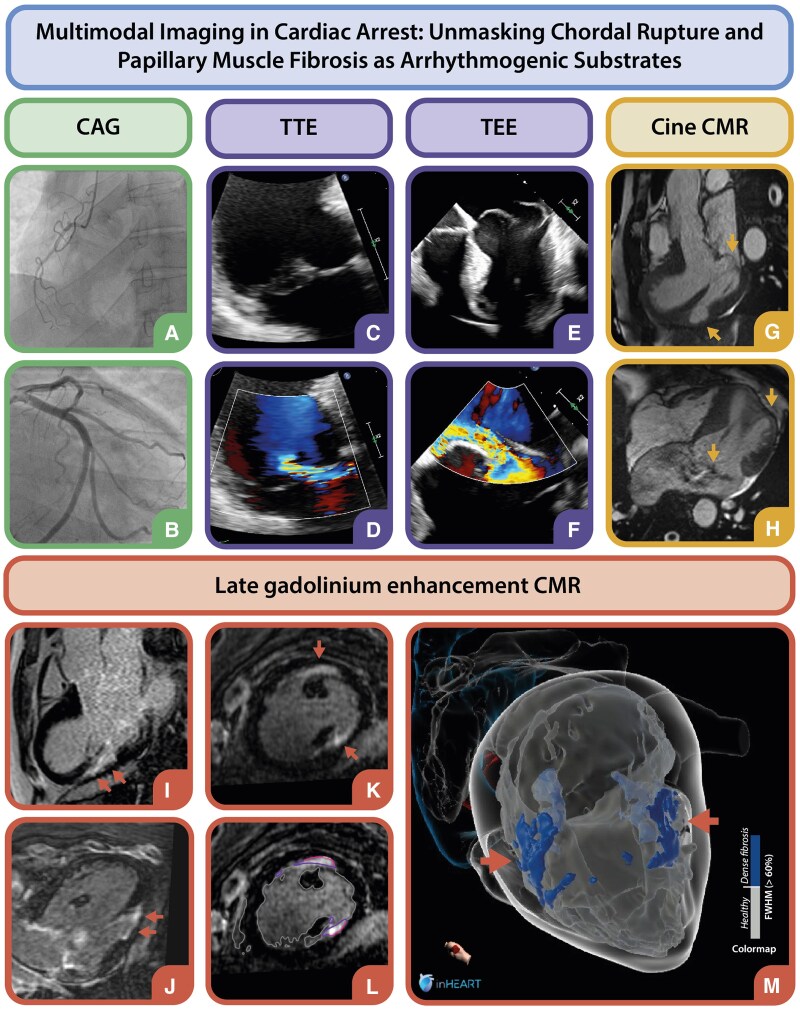


A 53-year-old male, previously evaluated for paroxysmal atrial fibrillation and mitral valve (MV) prolapse, was admitted after a witnessed out-of-hospital cardiac arrest due to ventricular fibrillation (VF). Paramedics delivered eight shocks for recurrent VF. Initial workup showed normal electrolytes and no evidence of ischaemia or genetic channelopathy. Coronary angiography excluded significant coronary artery disease (CAD) (*Panels A* and *B*). Transthoracic echocardiography, however, revealed a dilated left ventricle (LV) with mild dysfunction and severe mitral regurgitation (MR) due to posterior leaflet flail (*Panels C* and *D*). Trans-oesophageal echocardiography confirmed bi-leaflet myxomatous Barlow’s disease with anterior leaflet prolapse and posteromedial papillary muscle chordal rupture (*Panels E* and *F*).

To assess potential arrhythmogenic substrates, cardiac magnetic resonance imaging (CMR), including late gadolinium enhancement (LGE), was performed (see Supplementary data online). Cine CMR localized wall thinning at the myocardial insertion points of both papillary muscles (*Panels G* and *H*), with subendocardial fibrosis indicative of chronic fibrotic remodelling in the same areas (*Panels I*–*K*). Advanced 3D LGE visualization using inHEART software (inHEART Medical, Pessac, France) enabled high-quality mapping of fibrosis within the papillary muscle regions (*Panels L* and *M*).

The absence of significant CAD, combined with the observed subendocardial fibrosis pattern, suggests that chronic mechanical stress at the papillary muscle insertions, secondary to mitral valve prolapse, contributed to fibrotic remodelling and ultimately arrhythmogenesis. Due to severe primary MR with LV dilatation, evidence of mechanical fibrosis, and a dilated tricuspid valve (TV) annulus, MV and TV repair were performed, followed by implantable cardioverter-defibrillator placement for secondary prevention.

This case highlights the importance of multimodality imaging, including echocardiography and CMR, in evaluating structural heart disease and arrhythmic risk. It underscores the interplay between valvular pathology, mechanical stress, and fibrosis, highlighting the need for comprehensive imaging in risk stratification and clinical management.

Supplementary data are available at *European Heart Journal - Cardiovascular Imaging* online.

**Acknowledgements:** We thank the patient for consenting to share their case.

**Funding:** None.

**Data availability:** Available upon reasonable request.

## Supplementary Material

jeaf063_Supplementary_Data

